# Oxidized Ti Single Atoms and Co₃O₄ with Abundant Oxygen Vacancies Collaborating with Adjacent Pd Sites for an Efficient and Stable Oxygen Reduction Reaction

**DOI:** 10.1002/advs.202417789

**Published:** 2025-03-24

**Authors:** Hong‐Wei Chang, Thomas Yang, Che Yan, Po‐Han Chiu, Chi‐Ying Wu, Hung‐Wei Yen, Dinesh Bhalothia, Kaun‐Wen Wang, Po‐Chun Chen, Tsan‐Yao Chen

**Affiliations:** ^1^ Department of Engineering and System Science National Tsing Hua University Hsinchu 30013 Taiwan; ^2^ Department of Materials Science & Engineering National Taiwan University Taipei 10617 Taiwan; ^3^ Department of Materials and Mineral Resources Engineering National Taipei University of Technology Taipei 106344 Taiwan; ^4^ Advanced Research Center for Green Materials Science and Technology National Taiwan University Taipei 10617 Taiwan; ^5^ Department of Electronics and Communication Engineering Manipal University Jaipur Jaipur Rajasthan 303007 India; ^6^ Institute of Materials Science and Engineering National Central University Taoyuan City 32001 Taiwan; ^7^ Institute of Analytical and Environmental Science National Tsing Hua University Hsinchu 30013 Taiwan; ^8^ Institute of Nuclear Engineering and Science National Tsing Hua University Hsinchu 30013 Taiwan

**Keywords:** heterogenous catalyst, in situ XAS, oxygen reduction reaction, oxygen vacancies, single‐atom catalysts

## Abstract

The current study addresses these key issues by providing the ensemble sites and creating oxygen vacancies in the oxidized Ti‐single atoms. Here we report a novel heterogeneous catalyst comprising oxidized Ti‐single atoms uniformly coated on the cobalt‐oxide‐supported Pd nanoparticles (denoted as CP@Ti‐1), where the oxygen vacancies are introduced in oxidized Ti‐single atoms as well as cobalt‐oxide support. As‐developed CP@Ti‐1 catalyst demonstrates remarkable ORR activity with a mass activity (MA) of 9,725 mAmg_Ti_^−1^ at 0.85 V vs RHE and 1,244 mAmg_Ti_^−1^ at 0.90 V vs RHE in alkaline conditions. These values mark significant improvements over the commercial J.M.‐Pt/C catalyst, outperforming it by 145‐ and 50‐fold, respectively. Additionally, the CP@Ti‐1 catalyst shows exceptional durability, maintaining 100% of its initial performance after 20, 000 cycles of accelerated degradation test (ADT). In‐situ X‐ray absorption spectroscopy (XAS) analysis reveals that the catalyst design promotes synergistic interactions between oxygen vacancies in Ti/Co atoms and adjacent Pd domains, facilitating key reactions in ORR such as oxygen splitting and hydroxide ion formation, respectively, enhancing overall catalytic efficiency. These insights promise significant advancements in both scientific research and industrial applications of ORR catalysis.

## Graphical abstract

ORR pathways on the surface of CP@Ti‐1 catalyst, where oxygen vacancies in Co‐oxide and oxidized Ti single atoms promote the O_2_ splitting while adjacent Pd sites trigger the subsequent hydration step.

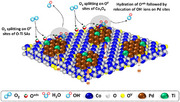

## 1 Introduction

Alkaline fuel cells are at the frontline for providing clean energy with net zero carbon footprints. However, their practical applications are severely hindered by the sluggish kinetics of oxygen reduction reaction (ORR), which needs to be boosted by deploying costly noble metal‐based catalysts based catalysts with high metal loading.^[^ ^1^, ^2^ ^]^ In this context, single‐atom catalysts (SAC)s have gained significant attention owing to their extraordinary properties such as efficient metal utilization and delineated active sites.^[^ ^3^, ^4^ ^]^ Despite potential advantages, SACs have certain limitations. For instance, the relatively low metal content in SACs and severe oxidation impact their catalytic performance.^[^ ^5^, ^6^ ^]^ Besides activity, the long‐term stability of SACs is hampered due to aggregation and other structural changes in the absence of steric protection.^[^ ^7^ ^]^ Moreover, the lack of ensemble reaction sites is a significant drawback of SACs, particularly in multistep electrochemical reactions like ORR.^[^ ^8^ ^]^ Consequently, developing effective strategies to surmount the limitations of SACs is imperative for realizing their industrial applications.

The aforementioned challenges have been addressed by multi‐directional approaches, where combining single atoms with nanoparticles, atomic clusters or other single atoms is at the forefront for augmenting their catalytic activity.^[^ ^9^, ^10^ ^]^ In such catalytic systems, two or more active sites simultaneously participate (direct or indirect) in catalyzing the reaction. In direct participation, adjacent active sites directly engage in the reaction process, each responsible for specific reaction steps.^[^ ^11^ ^]^ This dual‐site activation allows for simultaneous operation of all intermediate steps, culminating in a significant leap in the catalytic performance of electrocatalysts. On the other hand, unlike the first pathway, the Sabatier principle asserts dominance in indirect participation, where one active species induces modifications in the electronic and geometric structures of another, thereby optimizing the binding strength between reaction intermediates and the catalyst's surface.^[^ ^12^ ^]^ This optimized interaction results in an overall enhancement of catalytic performance. On top of that, our previous work combined both of these pathways, where Iridium (Ir)‐single atoms were decorated on the cobalt‐oxide‐supported Pd nanoparticles for ORR.^[^ ^13^ ^]^ In such a ternary catalyst, the Ir single‐atoms and Pd NPs directly participate in the ORR and synergistically facilitate the O_2_ splitting and hydration step, respectively. While the Co‐atoms indirectly participate and supply electrons to Ir‐single atoms for optimized binding energy with intermediates. A proper design it was, however, the Ir single atoms serve as the sole reaction sites for O_2_ splitting and therefore due to their ultra‐low Ir loading, these catalysts suffer from a shortage of sufficient reaction sites for effective O_2_ splitting, which remains a significant issue. In addition, the severe oxidation of Ir single atoms leads to suppressed O_2_ splitting kinetics. Furthermore, the Ir‐singel atoms were replaced by Pt‐dimers/trimers or atomic clusters in such structures, however, fewer reaction sites and the oxidation of Pt species resulted into suppressed catalytic activity and structure failure in stability tests, respectively.^[^ ^14^, ^15^ ^]^ To address the former issue (i.e., fewer reaction sites), recently published studies have demonstrated that oxygen vacancies (O^V^s) in transition metal oxides can serve as active sites for O_2_ splitting, therefore such metal oxides have been used as support materials for various single atom and atomic catalysts to increase the active sites for O_2_ splitting during ORR.^[^ ^16^, ^17^ ^]^ Despite numerous studies on noble metal and transition metal‐based SACs across different compositions and configurations, the second issue (i.e., oxidation) persists significantly.

This study strives to change the obstacle into an opportunity by generating O^V^s in oxidized single atoms. Herein, oxidized Ti‐single atoms are uniformly anchored on the surface of cobalt‐oxide‐supported Pd NPs and the O^V^s are created in the oxidized Ti‐single atoms as well as cobalt‐oxide support (henceforth denoted as CP@Ti‐1) for achieving high‐performance ORR. The CP@Ti‐1catalyst demonstrates superior performance compared to previously reported SACs, achieving a record‐high mass activity (MA) of 9725 mAmg_Ti_^−1^ at 0.85 V versus RHE and 1244 mAmg_Ti_^−1^ at 0.90 V versus RHE in alkaline ORR. These values represent a significant improvement, surpassing those of the commercial J.M.‐Pt/C catalyst (67  and 24.9 mA mg^−1^) by 145‐ and 50‐fold, respectively. Moreover, the catalyst exhibits exceptional durability under accelerated degradation testing (ADT), maintaining 100% of its pristine performance after 20, 000 cycles. The results of in situ XAS analysis reveal that such a design initiates the local synergy between O^V^s in Ti/Co atoms and neighboring Pd domains, respectively, facilitating the bond breaking (i.e., O_2_ splitting) and bond making (i.e., hydroxide ions formation) during ORR. We believe that this study introduces a highly efficient and economically competitive catalyst for ORR. Beyond its practical benefits, this work also enhances understanding of the relationship between catalyst structure and performance. Consequently, it promises significant advancements in both scientific understanding and industrial applications of ORR catalysis.

## 2 Results and Discussion

### 2.1 Physical Structure Inspection

**Figure 1:**
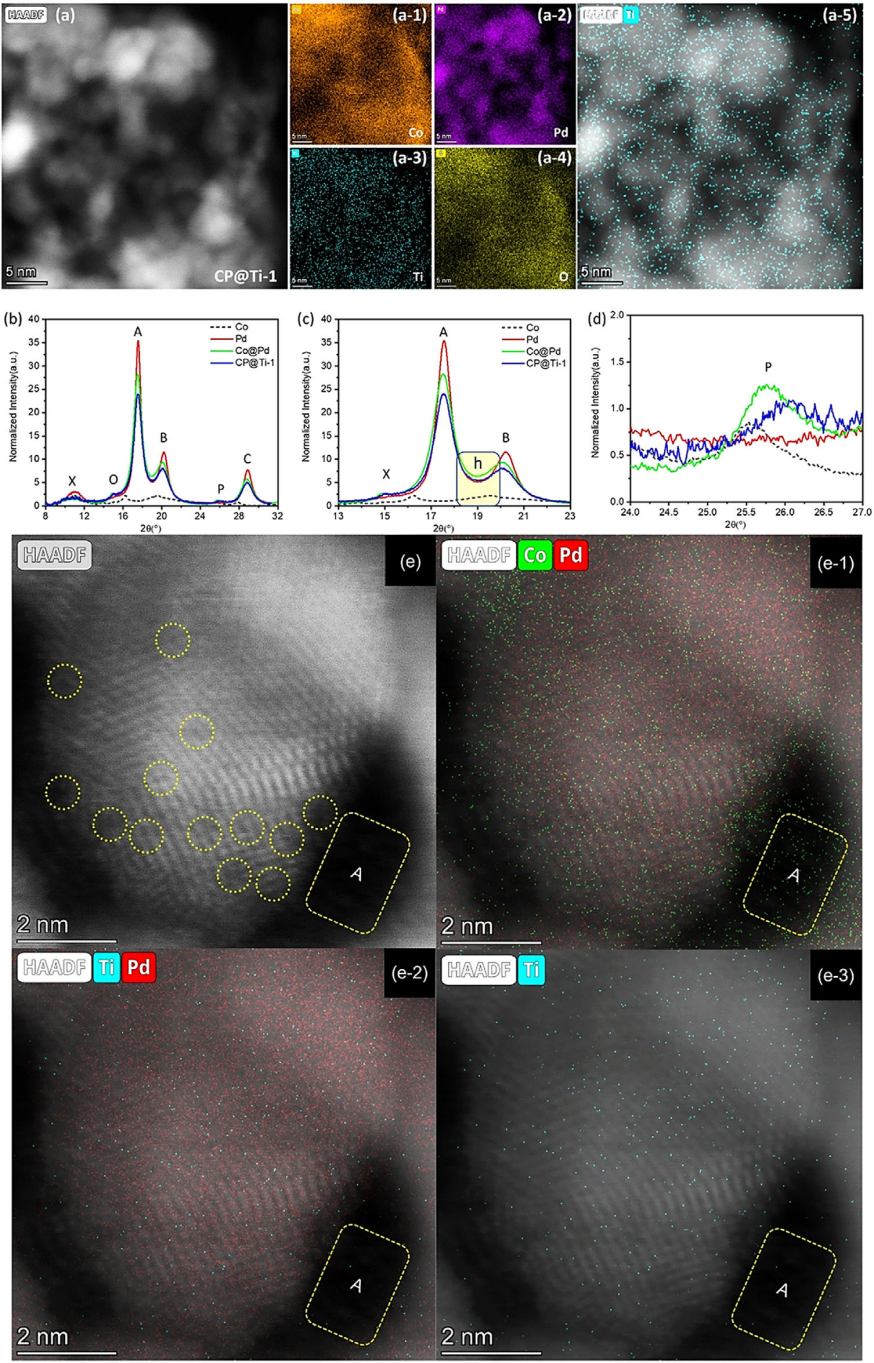
a) The HAADF‐STEM image and corresponding EDS elemental maps of CP@Ti‐1catalyst. b) X‐ray diffraction patterns of CP@Ti‐1catalyst compared with reference samples. The magnified sections of the XRD pattern are shown for the 2θ ranges of c) 13°–23° and d) 24°–27°. All diffraction patterns were recorded using incident X‐rays with an energy of 16 keV. e) The atomic resolution HAADF‐STEM image and corresponding EDS elemental maps for (e‐1) Co and Pd, (e‐2) Ti and Pd and (e‐3) Ti.

**Figure 2:**
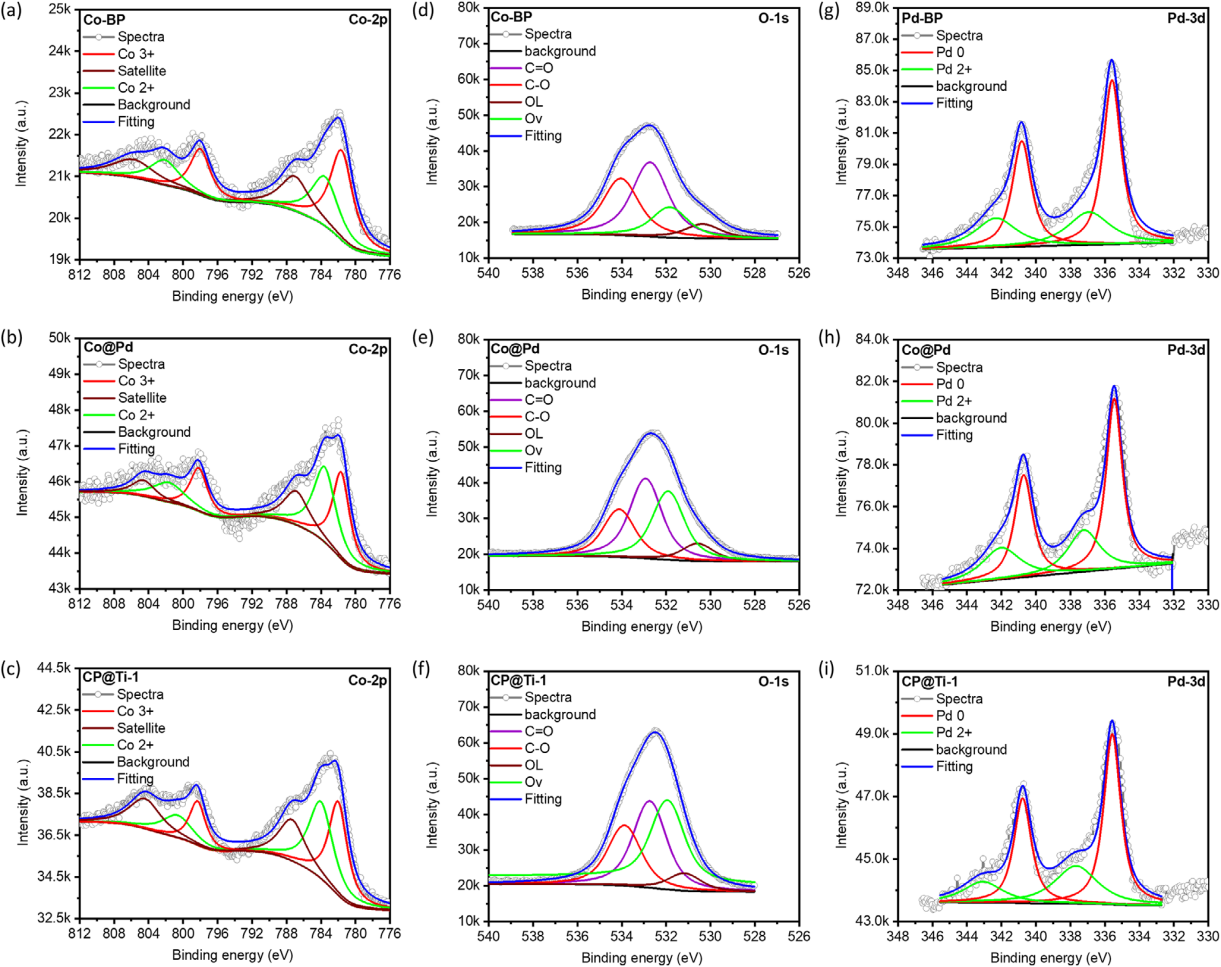
Comparative X‐ray photoelectron spectroscopy of a) Co‐BP, b) Co@Pd and c) CP@Ti‐1 at Co‐2p orbitals. d) Co‐BP, e) Co@Pd, and f) CP@Ti‐1 at O‐1s orbitals. g) Pd‐BP, h) Co@Pd, and i) CP@Ti‐1 at Pd‐3d orbitals.

**Figure 3:**
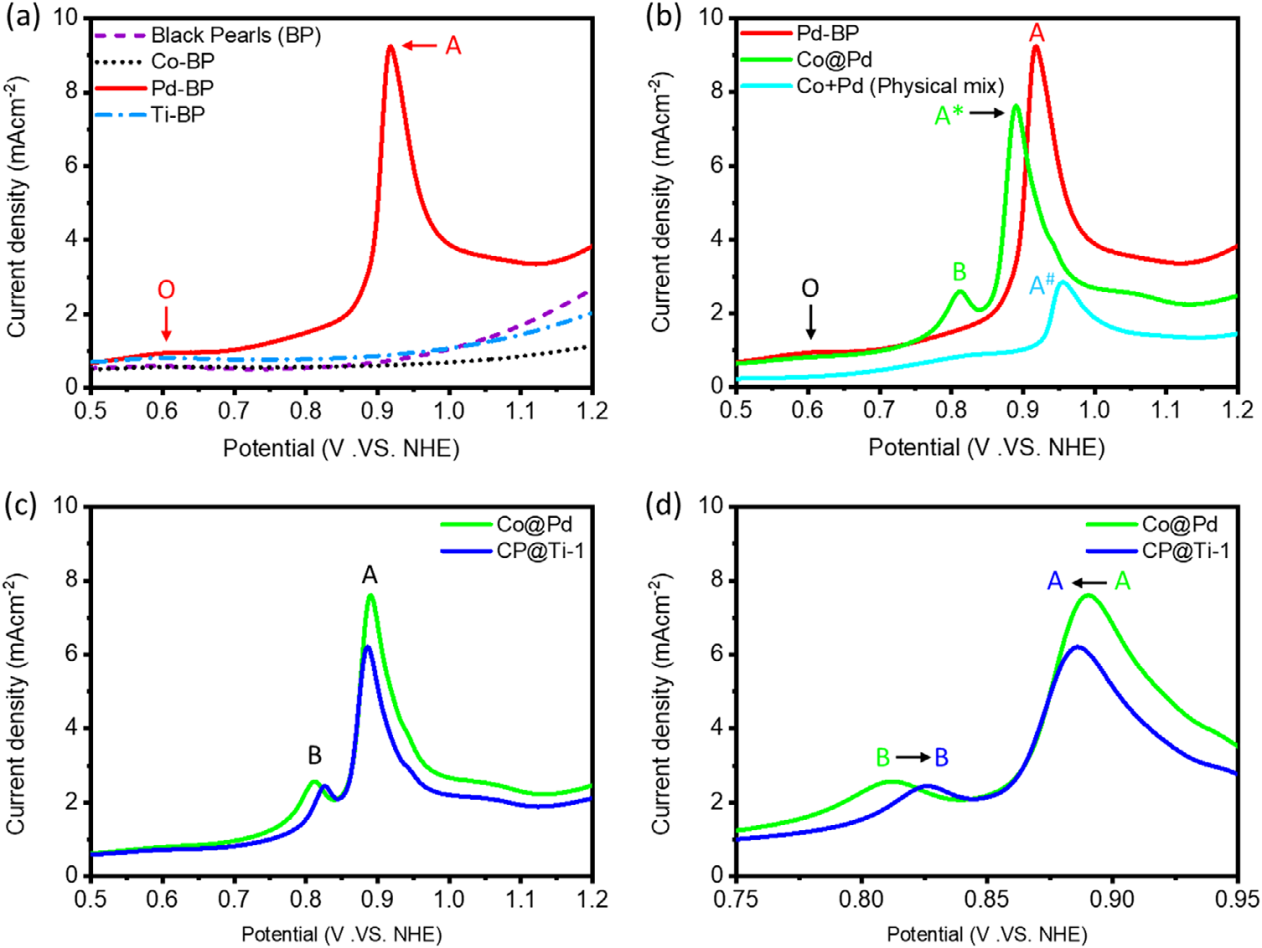
Comparative CO stripping curves of a) black pearls, Co‐BP, Pd‐BP and Ti‐P, b) Pd‐BP, Co@Pd and physical mixture of Co@Pd and c,d) Co@Pd and CP@Ti‐1 catalysts.

**Figure 4:**
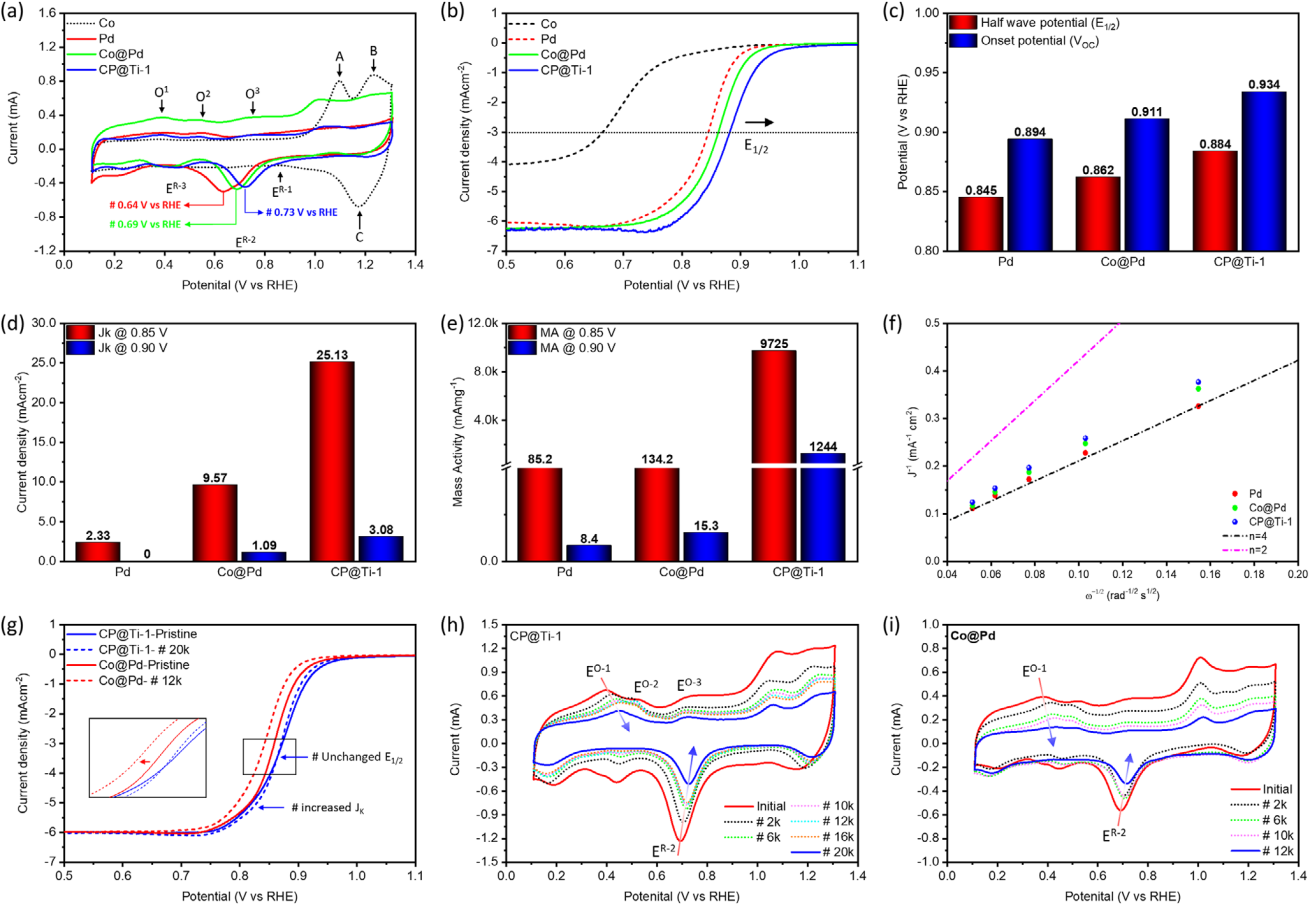
Electrochemical results. a) CV, (b) LSV curves, c) corresponding half‐wave potential (E_1/2_) and onset potential (Voc), d) kinetic current density, (e) ORR mass activity at 0.85  and 0.90 V versus RHE, f) Koutecky–Levich (K.L.) plot, g) LSV curves of CP@Ti‐1and Co@Pd catalysts at pristine and after ADT cycles, (h) CV curves of CP@Ti‐1catalyst at selected ADT cycles and (i) CV curves of Co@Pd catalyst at selected ADT cycles.

**Figure 5:**
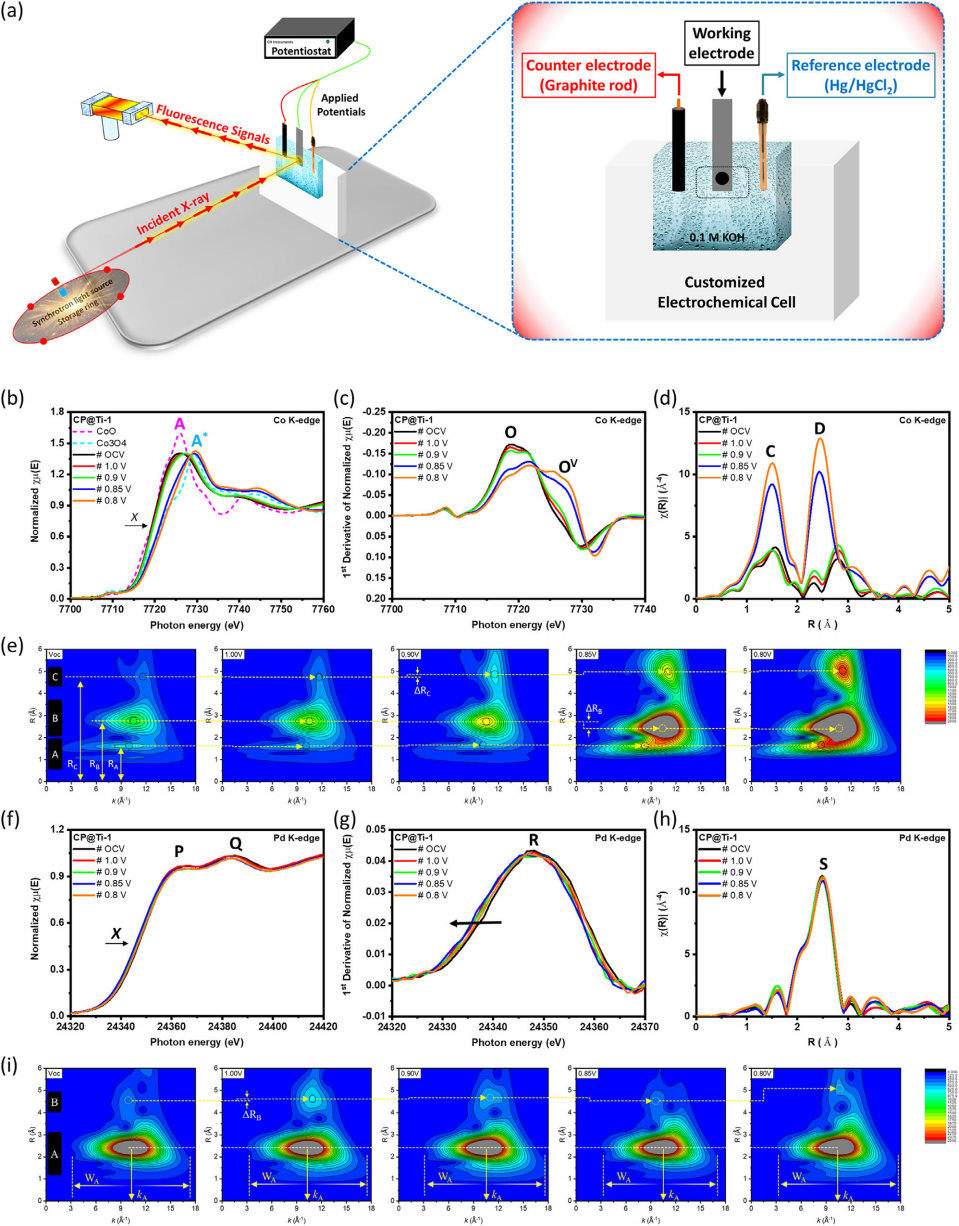
a) The schematic representation of the in situ XAS experiment setup and the electrochemical cell. b) In situ XANES c) 1st derivative, d) FT‐EXAFS spectra and e) wavelet patterns of the CP@Ti‐1 catalyst at the Co K‐edge. f) In situ XANES g) 1st derivative, h) FT‐EXAFS spectra and i) wavelet patterns of the CP@Ti‐1 catalyst at the Pd K‐edge.

Initially, the elemental composition of Co, Pd, and Ti in the CP@Ti‐1 catalyst was precisely quantified using inductively coupled plasma‐atomic emission spectroscopy (ICP‐AES). The analysis revealed their respective weight percentages as 16.2% for Co, 10.2% for Pd, and 0.66% for Ti. To elucidate the crystal structure of the CP@Ti‐1 catalyst, high‐angle annular dark‐field scanning transmission electron microscopy (HAADF‐STEM) combined with energy‐dispersive X‐ray spectroscopy (EDS) elemental mapping was employed. **Figure** **1**a presents the HAADF‐STEM image along with the corresponding EDS elemental maps. The distribution of Co (Figure 1a‐1) and Pd (Figure 1a‐2) confirms the formation of Pd nanoparticles supported on a Co‐oxide matrix. Furthermore, the successful incorporation of Ti single atoms within the CP@Ti‐1 catalyst is verified through the EDS elemental mapping of Ti (Figure 1a‐3, a‐5). Accordingly, the Ti‐single atoms are uniformly coated on the cobalt‐oxide‐supported Pd nanoparticles Given that Ti possesses the lowest atomic number (*Z* = 22) among the three elements in the system, its identification relies exclusively on EDS analysis. The HRTEM results of reference samples (i.e., Pd‐BP and Co@Pd) and CP@Ti‐1 are discussed in the Note S1 (Supporting Information).

Figure 1b presents a comparison of the X‐ray diffraction (XRD) patterns of the CP@Ti‐1 catalyst with reference samples. The characteristic diffraction peaks labeled as A, B, and C correspond to the (111), (200), and (220) planes of the metallic fcc Pd crystal, respectively. Additionally, peak M represents the characteristic signal of the (002) plane of the carbon support (BP‐2000), while peaks O and P correspond to the Co₃O₄ (311) plane and the Co₃O₄ (511) plane, respectively. For better clarity, magnified views of the peaks A/B (13–23°) and the peaks centered ≈(24–27°) are shown in Figure 1c,d, respectively. Given that the intensity of diffraction peaks primarily depends on factors such as crystallite size, phase purity, and atomic scattering power. The presence of Ti species on the Pd surface can disrupt the periodicity of the Pd crystal lattice, reducing the coherence length of X‐ray diffraction and thereby lowering peak intensity. Accordingly, compared to Co@Pd, the suppressed intensity of Pd diffraction peaks confirms that the Ti species are decorated on the Pd nanoparticles. Compared to Pd, the higher background (denoted by h in the yellow square) of Co@Pd indicates the higher surface roughness/defects in the Co@Pd.^[^ ^18^, ^19^ ^]^ More interestingly, the background of CP@Ti‐1 is again suppressed and aligns with Pd, suggesting a relatively smooth surface. These observations are in good agreement with the HRTEM results. The AC‐HAADF‐STEM image of CP@Ti‐1 is presented in Figure 1e, with corresponding atomic‐resolution elemental maps provided as follows: (**e‐1**) for Co and Pd, (**e‐2**) for Pd and Ti, and (**e‐3**) for Ti. Due to the significantly lower atomic number of Ti (22) compared to Pd (46), and because Ti atoms are uniformly dispersed on the nanoparticle surfaces rather than being aligned on the same crystallographic planes as the Pd atoms (a fact corroborated by the absence of any peak position shifts in the XRD diffraction patterns), it is exceedingly difficult to directly observe Ti atoms within the atomic lattice of the Pd NPs. In the image, the dashed yellow circles indicate positions of atomic vacancies within the Pd crystals. These vacancies arise because the uniformly deposited Ti atoms on the surface of the Pd crystals undergo oxidation upon exposure to air. This oxidation induces local lattice structure mismatches that generate strain fields and necessitate charge neutrality, leading to a disordered arrangement of Pd atoms in different regions of the NPs. Moreover, the presence of weak‐contrast, grayscale atomic clusters on the NP surfaces significantly diminishes the sharpness of the boundaries. This phenomenon clearly indicates that a high density of low‐atomic‐number oxides (i.e., Ti oxide), arranged in a nonuniform manner, coats the surfaces of the Pd NPs. Energy‐dispersive X‐ray spectroscopy (EDS) elemental mapping further confirms these observations. As shown in Figure (e‐1), the Co element is uniformly distributed across the image. In particular, the distribution of Co in region A does not overlap with that of Pd, confirming that Co is indeed uniformly dispersed on both the bottom and the surface of the Pd NPs enabling their collaboration with Ti atoms in ORR. The lack of contrast in the corresponding AC‐HADDF‐STEM image suggests that the Co, forming oxide structures, not only has a low atomic number but also exists in an amorphous state, thereby precluding the formation of a coherent electron diffraction signal. Figures (e‐2) and (e‐3) reveal that Ti atoms are uniformly distributed exclusively on the surface of the Pd NPs and are almost entirely absent on the Co oxide surfaces (as indicated by the dark contrast areas lacking atomic resolution). These results are in excellent agreement with the XRD analysis, which confirms that Ti exists predominantly as isolated single atoms on the surfaces of the Pd NPs. For further comparison, the analogous image analysis of the reference sample Co@Pd is provided in Figure S1 (Supporting Information). Similar to Figure 1e, region B in Figure S1 (Supporting Information) shows the presence of Co atoms, indicating that the Pd nanoparticles grow on the Co oxide surfaces. The yellow arrows denote that, in the absence of Ti modification, the atomic arrangement within the Pd nanoparticles is well‐ordered, and their surfaces do not exhibit scattered, low‐atomic‐number oxide structures. This observation further substantiates the atomic structure characterization of the CP@Ti‐1 sample as described in Figure 1e.

### 2.2 Confirmation of Oxygen Vacancies by XPS and EPR

The oxidation states and binding energies (BE) of the constituent elements along with the oxygen vacancies (O^V^) in the samples under investigation were analyzed using X‐ray photoelectron spectroscopy (XPS) at the Co‐2p, O‐1s, and Pd‐3d orbitals. In the Co‐2p spectrum (**Figure** **2**a–c), the deconvolution of the spectra reveals that the CP@Ti‐1 catalyst exhibits the highest Co^2^⁺/Co^3^⁺ ratios (0.94) as compared to Co‐BP (0.48) and Co@Pd (0.84), suggesting the highest extent of O^V^s in CP@Ti‐1 catalyst (**Table** **1**).^[^ ^20^ ^]^ Notably, oxygen vacancies in transition metal oxides typically lead to an increase in the lower oxidation state (e.g., Co^2^⁺) relative to the higher oxidation state (Co^3^⁺). This is because the removal of oxygen atoms from the lattice reduces the number of available O^2^⁻ ions required to stabilize Co^3^⁺, resulting in a partial reduction to Co^2^⁺.By calculating the Co^2^⁺/Co^3^⁺ ratio from the deconvoluted XPS spectra, the extent of oxygen vacancies has been confirmed. A higher Co^2^⁺/Co^3^⁺ ratio suggests a greater concentration of oxygen vacancies. Considering the quick oxidation of Ti single atoms, increased O^V^s in CP@Ti‐1 as compared to Co@Pd indicate the presence of abundant O^V^s in the oxidized Ti single atoms and consistently confirmed by the XPS spectra at Ti‐2p orbital of CP@Ti‐1 catalyst (Figure S6a, Supporting Information). The XPS spectra of the CP@Ti‐1 catalyst exhibit the presence of a Ti^3+^ oxidation state in significant amounts. In a perfect TiO₂ crystal, Ti typically exists in the Ti⁴⁺ oxidation state, as it is balanced with the oxygen ions (O^2^⁻). However, the reduction of Ti⁴⁺ to Ti^3^⁺ usually occurs when there is a deficiency of oxygen atoms, creating O^V^s to maintain charge neutrality.^[^ ^21^, ^22^ ^]^ The aforementioned scenarios are further confirmed by the XPS spectra at O‐1s orbitals (Figure 2d–f), where all the three samples including Co‐BP, Co@Pd, and CP@Ti‐1 exhibit a prominent O^V^ peak, confirming the presence of O^V^S in these samples. An even closer inspection of O^V^ peaks reveals that the CP@Ti‐1 catalyst exhibits the most intense O^V^ peak, which complementarily confirms the above‐discussed observations. The existence of O^V^S in Co@Pd and CP@Ti‐1 catalysts are further validated through variations in the spin density distribution under an applied magnetic field. Figure S6b (Supporting Information) shows the electron paramagnetic resonance (EPR) spectra of Co@Pd and CP@Ti‐1 catalysts, the CP@Ti‐1 catalyst exhibits the higher peak intensity as compared to Co@Pd, confirming the presence of O^V^S in decorated Ti species. Furthermore Figure 2g–i present the XPS spectra of Pd‐BP, Co@Pd, and CP@Ti‐1 catalysts at Pd‐3d orbitals, respectively, where the corresponding oxidation states and binding energies are summarized in Table 1. Compared to Pd‐BP, the Co@Pd and CP@Ti‐1 catalysts show the increased oxidation of Pd which can be attributed to the presence of Co‐oxide and additional oxidized Ti single atoms. More importantly, the increased binding energy of Co (compared to Co‐BP) and reduced binding of Pd^0^ (compared to Pd‐BP) indicates the electron localization from Co‐to‐Pd in the Co@Pd catalyst. Unsurprisingly, along with the Co, the Pd also exhibits increased binding energy for CP@Ti‐1 as compared to Co@Pd. Such a scenario suggests the further electron relocation from Pd to O^V^ sites of oxidized Ti single atoms due to the high electronegativity of O^V^S.

**Table 1:** XPS determined elemental chemical states and binding energies.

	Oxidation State					Ratio	Binding Energy [eV]			
	Pd 0	Pd ^2+^	Co ^2+^	Co ^3+^	O_V_	Co^2+^/Co^3+^	Pd ^0^	Pd ^2+^	Co ^2+^	Co ^3+^
Co‐BP	N/A		32.5	67.5	28.65	0.48			783.68	781.65
Pd‐BP	67.59	32.41	N/A				335.57	336.84	N/A	
Co@Pd	63.45	36.55	45.8	54.2	46.98	0.84	335.39	337.17	783.79	781.75
CP@Ti‐1	61.31	38.69	48.6	51.4	57.58	0.94	335.55	337.66	784.05	781.91

### 2.3 CO‐Stripping Analysis

CO‐stripping analysis was utilized to determine the surface chemical properties of CP@Ti‐1catalyst and reference samples. Specifically, the positions of the oxidation peaks of adsorbed CO (CO^ads^) in the CO‐stripping curve offer insights into the required potential for CO oxidation.^[^ ^23^ ^]^ Additionally, the area under the CO oxidation peak reflects the density of surface‐active sites that have undergone CO chemisorption.^[^ ^23^ ^]^ As shown in **Figure** **3**a, the absence of current responses in the CO stripping curves of the substrate (i.e., black pearls), Co, and Ti nanoparticles suggest their inert behavior toward CO molecules. On the other hand, the BP‐supported Pd NPs (i.e., Pd‐BP) exhibit a distinct CO‐oxidation peak (A) at ≈0.92 V versus NHE, corresponding to the compact Pd (111) facet.^[^ ^24^ ^]^ Additionally, a suppressed peak (O) at the lower potential region refers to the surface defects, confirming the presence of surface defects in Pd nanoparticles.^[^ ^25^ ^]^ Figure 3b compares the CO stripping curves of Pd NPs with Co@Pd and the physical mixture of Co+Pd. Accordingly, compared to the main CO^ads^ oxidation peak of Pd nanoparticles (i.e., peak A), an offset of −30 mV (peak A^*^) for Co@Pd suggests a significantly lower energy barrier for CO^ads^ oxidation on the surface of Co@Pd and can be attributed to the charge localization on Pd surface from Co‐domains (consistent with XPS observations). More importantly, the rise of an additional peak B at the lower potential (≈0.810 V vs NHE) corresponds to the CO^ads^ oxidation at the low energy barrier reaction sites. Considering the absence of this peak in Pd nanoparticles and the physical mixture of Co+Pd, it can be assigned to the Co‐to‐Pd interface. In addition, compared to Pd NPs, the significant suppression in peak O indicates reduced surface defects in Co@Pd. Meanwhile, the physical mixture of Co+Pd exhibits a suppressed peak A^#^ at the highest potential, suggesting the highest energy barrier for CO^ads^ oxidation. Furthermore, the CO stripping curve of CP@Ti‐1catalyst is compared with Co@Pd in Figure 3c, while the zoom in region is shown in Figure 3d. Notably, compared to Co@Pd, the significant suppression of the main CO^ads^ oxidation peaks for CP@Ti‐1catalyst confirms the reduced surface Pd sites in this sample due to surface coverage via Ti‐atoms, as consistently proved by their inert nature toward CO molecules in the CO‐stripping curve of Ti nanoparticles.^[^ ^13^ ^]^ More importantly, a shift of the main oxidation peak “A” to the lower potential confirms the increased CO‐tolerance of CP@Ti‐1 catalyst as compared to Co@Pd.^[^ ^26^, ^27^ ^]^ Moreover, the shift of peak B to higher potentials indicates that along with the surface of Co@Pd, Ti‐atoms are also decorated at the Co‐to‐Pd interface.

### 2.4 Performance Analysis in Oxygen Reduction Reaction

**Figure** **4**a shows the cyclic voltammetry (CV) curves of CP@Ti‐1catalyst and control samples. For Co nanoparticles, the absence of current signals up to 1.0 V versus RHE suggests the severe oxidation of Co nanoparticles and therefore exhibits the chemically inert behavior for ORR. Meanwhile, the two peaks (A and B) in the forward sweep and peak C in the reverse sweep can, respectively be attributed to the adsorption and subsequent reduction of oxygenated species from the Co surface at higher potentials. The Pd nanoparticles exhibit a smeared peak profile (indicating high H^+^ ions adsorption affinity of Pd) in the underpotential deposition region of hydrogen (H_UPH_) and an obvious oxide reduction peak E^R‐2^ at 0.64 V versus RHE.^[^ ^28^ ^]^ Notably, the Co@Pd and CP@Ti‐1catalysts exhibit similar peak profiles to Pd (peaks O^1^, O^2^, O^3^ in forward sweep and oxide reduction peak E^R‐2^ in reverse sweep) and Co (peaks A/B in forward sweep and peak C in reverse sweep), respectively in lower and higher potential regions, confirming the formation of Pd nanoparticles over Co‐oxide support in these samples. Moreover, compared to Pd and Co@Pd, the highest positive shift of the oxide reduction for the CP@Ti‐1catalyst suggests the lowest energy barrier for ORR on its surface.^[^ ^13^ ^]^ More interestingly, an additional shoulder peak E^R‐1^ (≈0.856 V vs RHE) is observed for the CP@Ti‐1 catalyst, which corresponds to the oxide reduction from Ti‐sites with lower energy barriers as compared to that of adjacent Pd‐sites.

The catalytic activities of CP@Ti‐1catalyst and control samples were elucidated by using linear sweep voltammetry (LSV), as shown in Figure 4b. For a fair comparison, the ORR performance of commercial J.M.‐Pt/C catalyst with 20 wt.% of Pt and the control samples (i.e., Co, Pd, and Co@Pd) were assessed under similar conditions. Notably, the highest half‐wave (E_1/2_) and onset (V_OC_) potentials (Figure 4c) for the CP@Ti‐1 catalyst indicate the highest reaction kinetics for ORR with the lowest energy barrier, which is in good agreement with the position of the oxide reduction peak (E^R‐2^) in CV curve (Figure 4a). Next, the mass activities (MA)s of the CP@Ti‐1catalyst and control samples were obtained at 0.85 and 0.90 V versus RHE relative to Ti‐loading. Herein it is worth noticing that the kinetic current density (J_k_) (Figure 4d) of Co@Pd is first deducted from the J_k_ of the CP@Ti‐1catalyst for the calculation of MA values to ensure that MA is completely dominated by the decorated Ti single atoms (detailed calculations procedure for MA is provided in Note S2, Supporting Information). Accordingly, the CP@Ti‐1 exhibits exceptional MA values of 9725 and 1244 mAmg_Ti_^−1^ at 0.85  and 0.90 V versus RHE, respectively (Figure 4e). Given that the ORR performance matrices (i.e., E_1/2_, V_OC_, J_k_, and MA) of Co@Pd are far below than CP@Ti‐1catalyst, confirming that the ORR kinetics of the CP@Ti‐1 catalyst is dominated by the decorated Ti‐single atoms. The ORR performance of the CP@Ti‐1catalyst is further compared with commercial J.M.‐Pt/C in Figure S7 (Supporting Information), where the MA values of the CP@Ti‐1catalyst outperform the commercial J.M.‐Pt/C by 145‐ and 50‐fold, respectively at 0.85  and 0.90 V versus RHE. Ultimately, the ORR performance metrics of the CP@Ti‐1 catalyst are compared with those reported in the literature (Table S1, Supporting Information), underscoring the superiority of CP@Ti‐1 over other catalysts. The Koutecky–Levich (K.L.) plots confirm the 4‐electron transfer pathway for the catalysts under investigation (Figure 4f).

Inspired by the high ORR performance of the CP@Ti‐1 catalyst, its long‐term stability in ORR is evaluated by an accelerated degradation test (ADT). As shown in Figure 4g, the unchanged E_1/2_ for the CP@Ti‐1catalyst after 20000 ADT cycles confirms its high stability. On the other hand, Co@Pd shows a negative offset of −16 mV just after 12000 ADT cycles. Figure 4h,i, respectively, show the CV curves of CP@Ti‐1 and Co@Pd catalysts at selected ADT cycles. Herein it's worth noticing that the Pd‐oxide reduction peaks (E^R‐2^) exhibit similar behavior (a progressive upshift in position and intensity) with increasing ADT cycles in both samples, suggesting a similar chemical reaction (i.e., removal of surface oxide) on the Pd surface. Considering such a scenario, it can be concluded that the decorated Ti‐single atoms are dominating reaction sites in the CP@Ti‐1 catalysts during ORR.^[^ ^13^ ^]^

### 2.5 Unveiling ORR Pathways via In Situ XAS Analysis

The ORR pathways on the surface of the CP@Ti‐1catalyst were determined through in situ XAS analysis at the Co, Pd, and Ti K‐edges. As shown in **Figure** **5**a, Fluorescence‐mode in situ XAS spectra were obtained using a custom‐built electrochemical cell coupled with a CH Instruments Model 600B potentiostat, which featured a three‐electrode configuration. The in situ X‐ray absorption near‐edge structure (XANES) spectra at the Co K‐edge for the CP@Ti‐1 catalyst under potential‐driven conditions are presented in Figure 5b. The inflection point (X), which corresponds to peak O in the first derivative curve (Figure 5c), appears at a similar position to that of CoO. This observation indicates that cobalt primarily exists in the form of CoO under open‐circuit voltage (OCV) conditions and within the lower potential range of 1.0 –0.9 V versus RHE in the CP@Ti‐1 catalyst. Notably, in the typical Co K‐edge spectrum, the white line intensity (H_A_) and the position of inflection point (X) correspond to the surface chemical adsorption of oxygenated species and the oxidation state of targeted atoms, respectively.^[^ ^16^ ^]^ Interstingly, the CP@Ti‐1 catalyst exhibits suppressed H_A_ as compared to CoO in this applied potential range, indicating the presence of severe O^V^S in the Co domains of the CP@Ti‐1 catalyst. Further increasing the potential from 0.9 V to 0.85/0.8 V leads to the positive shift of inflection point (similar to Co_3_O_4_), suggesting the increased oxidation state of Co. Along with the presence of shoulder peak (O^V^) in the first derivative curve (Figure 5c) at the applied potential range of 0.85 –0.8 V, such an increased oxidation state confirms that O^V^S in the Co domains promotes the O_2_ splitting (O^2^ → 2O^ads^) step during ORR on the surface of CP@Ti‐1 catalyst.^[^ ^16^ ^]^ Figure 5d shows the FT‐EXAFS spectra of the CP@Ti‐1 catalyst at the Co K‐edge, where peaks labeled as “C” and “D” are contributions of the Co‐O bond pairs in the 1st and 2nd coordination shell around Co atoms.^[^ ^16^ ^]^ Consistent with the XANES (Figure 5b) and first derivative curves (Figure 5c), the intense Co‐O peaks at the applied potential range of 0.85 –0.8 V in the FT‐EXAFS spectra, confirming the direct participation of Co domains in the O_2_ splitting kinetics. These observations are complementarily confirmed by the wavelet transform (WT) patterns. The WT patterns offer the ability to distinguish backscattering atoms by providing both k‐space and radial distance resolution.^[^ ^29^ ^]^ As a result, WT analysis of the FT‐EXAFS spectra at the Co K edge is conducted to further verify the contribution of Co domains in ORR. As shown in Figure 5e, the progressively increasing intensities of maxima B (2.9 Å) and C (4.85 Å) (corresponds to the Co‐O bond pair in the 1st and 2nd coordination shells) again confirms that Co domains are proactively favored the O_2_ splitting kinetics during ORR. The in situ XANES spectra of the CP@Ti‐1 catalyst at the Pd K‐edge (Figure 5f) reveal that the inflection point X (corresponding to peak R in the 1st derivative curve (Figure 5g) occurs at the highest energy value under OCV conditions, suggesting the presence of oxygenated species (OH^−^ ions) on the Pd surface. Further increasing the potential up to 0.8 V versus RHE leads to the progressive shift of inflection point X to the lower energy values, suggesting the decreasing oxidation state (i.e., removal of oxygenated species) from the surface. Given that the Pd is chemically stable due to its noble nature, which resists oxidation in the operating potential range of the ORR (0.6–1.0 V vs RHE). Within this range, Pd primarily exists in its metallic (Pd^0^) state or a weakly oxidized state due to hydroxide ions adsorption (i.e., Pd–OH).^[^ ^30^ ^]^ These observations integrally confirm that the O^ads^ atoms (from Co‐domains) relocate on the Pd surface and reduce to OH^−^ ions due to the presence of a high density of electrons (confirmed by XPS in the former section) followed by desorption of the same under potential driven conditions. Figure 5h,i, respectively, show the FT‐EXAFS spectra and corresponding WT patterns of the CP@Ti‐1 catalyst at Pd K‐edge. Accordingly, the highest intensities of peak S in the FT‐EXAFS spectra and maxima A in the WT patterns complementarily confirm the presence of OH^−^ ions under OCV conditions. Meanwhile compared to the OCV state, the progressively suppressing intensities of these peaks indicate the removal of OH^−^ ions under potential‐driven conditions. The in situ XAS spectra of Co@Pd at Co and Pd K‐edges have been shown in Figure S8 (Supporting Information) for reference.

**Figure 6:**
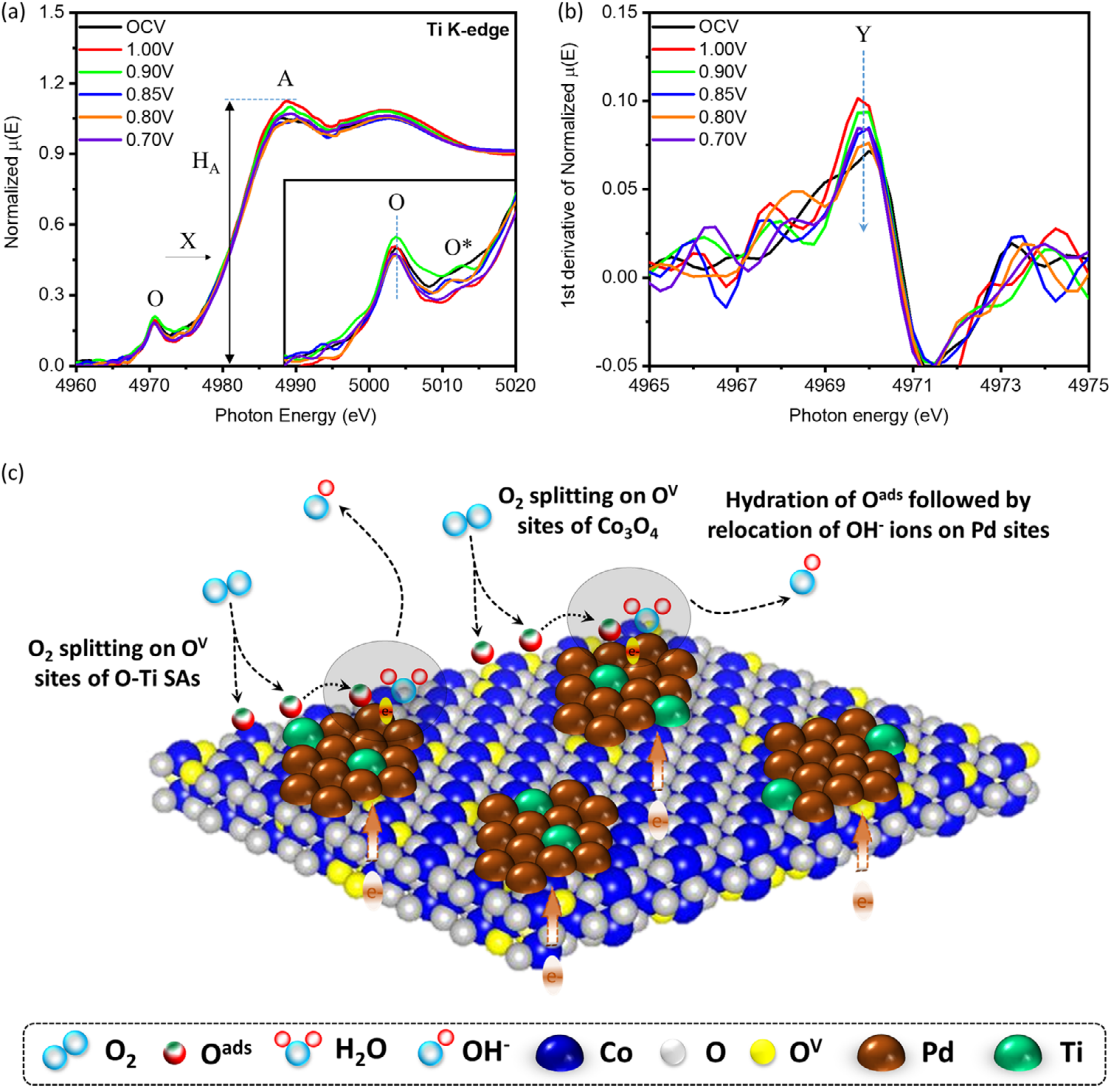
a) In situ XANES and b) 1st derivative spectra of the CP@Ti‐1 catalyst at the Ti K‐edge. c) The ORR pathways on the surface of CP@Ti‐1 catalyst.

Finally, the in situ XANES spectra of the CP@Ti‐1 catalyst at the Ti K‐edge have been analyzed to elucidate the role of Ti‐single atoms during ORR. As shown in **Figure** **6**a, the similar position of inflection point (X) (corresponds to the peak Y in the 1st derivative spectra (Figure 6b) suggests the unchanged chemical (oxidation) state of Ti atoms under potential‐driven conditions. On the other hand, the white line intensity (H_A_) of the CP@Ti‐1 catalyst dramatically enhanced under the applied potential of 1.0 V‐0.9 V versus RHE as compared to OCV conditions, suggesting the presence of adsorbed oxygen (O^ads^) on the Ti‐atoms under these applied potentials and can be attributed to the O_2_ splitting (O^2^ → 2O^ads^) step on the Ti‐atoms during ORR.^[^ ^15^ ^]^ Further raising the potential up to 0.8 V versus RHE leads to the suppressed H_A_ (similar to OCV conditions), suggesting the relocation of O^ads^ to the adjacent Pd sites. These observations are in good agreement with the above‐discussed Pd K‐edge results. These scenarios are complementarily confirmed by the pre‐edge analysis (shown in the inset of Figure 6a). The splitting of pre‐edge peak O to O* corresponds to transforming the Ti‐O site from a distorted tetrahedral to an octahedral Ti‐O site by increasing the applied potential from OCV to 0.80 V (V vs RHE). When the potential changes to 0.70 V (V vs RHE), the absence of peak X* indicates the formation of a tetrahedral Ti‐O4 site. In the absence of chemical shift (see the position of inflection peak X in Figure 6a), these scenarios reveal that Ti‐Ox are empty sites for splitting/storing the O^ads^. By cross‐referencing the results of in situ XAS analysis at Co, Pd, and Ti K‐edge, the ORR pathways are determined and presented in Figure 6c.

## 3 Conclusion

In conclusion, this study highlights the development of a novel heterogeneous catalyst, CP@Ti‐1, featuring oxidized Ti‐single atoms uniformly coated on cobalt‐oxide‐supported Pd nanoparticles, with oxygen vacancies introduced in both the oxidized Ti‐single atoms and the cobalt‐oxide support. The CP@Ti‐1 catalyst exhibits remarkable oxygen reduction reaction (ORR) activity, achieving mass activities of 9725 mA mgTi⁻¹ at 0.85 V and 1244 mA mgTi⁻¹ at 0.90 V versus RHE under alkaline conditions. These values significantly surpass the performance of the commercial J.M.‐Pt/C catalyst by 145‐fold and 50‐fold, respectively. Furthermore, the CP@Ti‐1 catalyst demonstrates exceptional durability, retaining its full performance after 20000 cycles of accelerated degradation testing. In situ X‐ray absorption spectroscopy (XAS) reveals that the synergistic interactions between oxygen vacancies in Ti/Co atoms and adjacent Pd domains enhance key reaction steps in ORR, such as oxygen splitting and hydroxide ion formation, thereby boosting overall catalytic efficiency. This work not only establishes a cost‐effective and highly efficient ORR catalyst but also deepens the understanding of the relationship between catalyst structure and performance, paving the way for future advancements in ORR catalysis for both research and industrial applications.

## 4 Experimental Section

### Preparation of Catalysts

The ternary heterogeneous catalyst comprising uniform coating of Ti‐single atoms on the cobalt‐oxide‐supported Pd nanoparticles was synthesized using a carefully controlled approach that involved ion chemisorption followed by quick reduction and ambient annealing. The size and dispersion of Ti‐atoms were precisely managed by adjusting the molar ratios of Ti/Pd and controlling the reaction time during chemisorption and reduction of Ti^4+^ ions. In this study, the acid‐treated carbon black pearls (BP‐2000) were used as a substrate to grow the catalyst. The acid treatment involved immersing the black pearls in H_2_SO_4_ at 80 °C for 6 h, followed by thorough rinsing with deionized (D.I.) water until the pH of the rinsing water reached 6.0. In the first step, 6 g of 1.0 wt.% BP solution in D.I. water and Polyvinylpyrrolidone (PVP) (i.e., 60 mg of BP) is dispersed in 3.06 g of an aqueous 0.1 m cobalt (II) chloride (CoCl_2_, 99%, Sigma–Aldrich Co.) solution and stirred at 600 rpm for 4 h followed by the reduction of Co^2+^ ions via sodium borohydride (NaBH_4_; 99%, Sigma–Aldrich Co.) (0.11 g of NaBH_4_ in 5.0 mL of D.I. water solution) forming metastable Co metal NPs, which subsequently oxidized to Co‐oxide (CoOx). Herein the weight ratio of Co/BP is 30 wt.%. Next, 3.06 g of a Pd precursor solution containing 0.306 mmoles of Pd metal ions (0.1 m) was added to the CoOx‐BP solution to grow Pd NPs on the CoOx domains (designated as Co@Pd). Here it is worth mentioning that the Pd^2+^ ions were reduced by excess NaBH_4_ added in the previous step. In this way, the galvanic replacement reaction between Pd^2+^ and Co^0^ was restricted. After synthesizing Co@Pd NPs, a Ti‐precursor solution was added to decorate the Ti‐single atoms on the surface of Co@Pd. Prior to Ti‐single atoms decoration, the Co@Pd NPs underwent ultrasonication to create surface defects, facilitating the surface anchoring of Ti species. To control the size and distribution of Ti decoration, the molar ratio of Ti/Pd was set to 0.05 (i.e., 1 wt.% of Ti). Throughout this article, the Co@Pd NPs decorated with 1 wt.% of Ti are referred to as CP@Ti‐1.

### Physical Characterizations

The atomic composition analysis of the catalysts was conducted using inductively coupled plasma‐atomic emission spectrometry (ICP‐AES) with a Jarrell‐Ash ICAP 9000 instrument. For structural elucidation, a combination of microscopy and X‐ray spectroscopy techniques was employed. The high‐angle annular dark‐field scanning transmission electron microscopy (HAADF‐STEM) images and high‐resolution transmission electron microscopy (HRTEM) images were collected by Talos F200X (Thermo‐Fisher) at the Electron Microscopy Centre in the National Sun Yat‐sen University, Taiwan. The aberration‐corrected HAADF‐STEM (AC‐HADDF‐STEM) images and corresponding Energy‐dispersive X‐ray spectroscopy (EDS) for elemental mappings are acquired by Spectra 300 (Thermo‐Fisher) at Instrumentation Center in National Taiwan University. X‐ray diffraction (XRD) patterns were obtained at beamline BL‐01C2 of the National Synchrotron Radiation Research Center (NSRRC), Taiwan, using an incident X‐ray wavelength of 0.6888 Å (18.0 KeV). X‐ray absorption spectroscopy (XAS) was employed to examine electronic states and atomic arrangements. XAS spectra at Co K‐edge, Pd K‐edge, and Ti K‐edge were measured in fluorescence mode at beamlines BL‐17C and 01C1 of NSRRC, Taiwan. X‐ray photoelectron spectroscopy (XPS) analysis was conducted at beamline BL‐24A1 of NSRRC, Taiwan, to investigate oxidation states and surface compositions. Proposed oxygen reduction reaction (ORR) pathways were validated through in situ XAS inspections, performed using a custom single‐compartment Teflon electrochemical cell at the SP8‐12B2 beamline of Spring‐8, Japan.

### Electrochemical Analysis

Electrochemical measurements were conducted at a constant room temperature of 25 ± 1 °C using a potentiostat (CH Instruments Model 600B, CHI 600B) equipped with a three‐electrode system. For the ORR experiment, a catalyst slurry was prepared by dispersing 5 mg of catalyst powder in 1.0 mL of isopropanol (IPA) containing 50 µL of Nafion‐117 (99%, Sigma‐Aldrich Co.) as a conducting binder. Prior to the ORR test, the mixture underwent 30 min of ultrasonication. During the ORR test, 10.0 µL of the catalyst slurry was drop‐cast onto a glassy carbon rotating disk electrode (RDE) with an area of 0.196 cm^2^, serving as the working electrode. A Hg/HgCl_2_ electrode, referenced to 0.242 V versus the reversible hydrogen electrode (RHE) and saturated in KCl aqueous solution, was used as the reference electrode. A graphite rod was employed as the counter electrode to prevent Pt contamination. Cyclic voltammetry (CV) and linear sweep voltammetry (LSV) data were acquired using scan rates of 0.02 V s⁻¹ and 0.001 V s⁻¹, respectively. The potential ranges were 0.1  to 1.3 V versus RHE for CV and 0.4  to 1.1 V versus RHE for LSV, in a 0.1 m KOH aqueous alkaline electrolyte solution (pH 13). During LSV, the rotation rate varied from 400 to 3600 rpm. CV experiments were conducted under a nitrogen (N_2_) atmosphere, while LSV experiments were carried out under an oxygen (O_2_) atmosphere. The electrochemical stability of the catalyst was evaluated through an accelerated durability test (ADT) conducted within a potential range of 0.5 –1.0 V versus RHE. The ADT involved a scan rate of 0.05 V s⁻¹ in an O_2_ atmosphere across multiple cycles to assess the catalyst's performance over time.

For CO‐stripping analysis, a glassy carbon electrode, Pt wire and Ag/AgCl electrode were used as the working, counter and reference electrodes, respectively. The adsorption of CO on the surface of the catalyst was performed by purging CO into 0.5 m H_2_SO_4_ at 0.05 V (vs NHE) for 20 min. Then the CO stripping voltammetry was measured between −0.10 and 1.20 V (vs NHE) in N_2_ saturated 0.5 m H_2_SO_4_ solution at a scan rate of 50 mVs^−1^.

## Conflict of Interest

The authors declare no conflict of interest.

## Supporting information



Supporting Information

## Data Availability

The data that support the findings of this study are available from the corresponding author upon reasonable request.
