# Supplementary material for: Oxidized Ti Single Atoms and Co₃O₄ with Abundant Oxygen Vacancies Collaborating with Adjacent Pd Sites for an Efficient and Stable Oxygen Reduction Reaction
Source: Adv Sci (Weinh). 2025 Mar 24;12(19):2417789. doi: 10.1002/advs.202417789 (PMC12097026; doi:10.1002/advs.202417789)
Supplement: Supplementary file 1 — Supporting Information [file ADVS-12-2417789-s001.docx]

# **Supplementary information for**

**Oxidized Ti Single Atoms and Co₃O₄ with Abundant Oxygen Vacancies Collaborating with Adjacent Pd Sites for an Efficient and Stable Oxygen Reduction Reaction**

Hong-Wei Chang,^a^ Thomas Yang,^a^ Che Yan,^a^ Po-Han Chiu,^b^ Chi-Ying Wu,^c^ Hung-Wei Yen,^c^ Dinesh Bhalothia,^d,*^ Kaun-Wen Wang,^e^ Po-Chun Chen,^c*^ and Tsan-Yao Chen^a,f,g*^

Affiliations:

^a.^ Department of Engineering and System Science, National Tsing Hua University, Hsinchu 30013, Taiwan

^b.^ Department of Materials Science & Engineering, National Taiwan University, Taipei 10617 Taiwan

^c.^ Department of Materials and Mineral Resources Engineering, National Taipei University of Technology

^d.^ Department of Electronics and Communication Engineering, Manipal University Jaipur, Rajasthan 303007, India

^e.^ Institute of Materials Science and Engineering, National Central University, Taoyuan City 32001, Taiwan

^f.^ Institute of Analytical and Environmental Science, National Tsing Hua University, Hsinchu 30013, Taiwan

^g.^ Institute of Nuclear Engineering and Science, National Tsing Hua University, Hsinchu 30013, Taiwan

*Corresponding Author(s):

Prof. Dinesh Bhalothia

Department of Electronics and Communication Engineering,

Manipal University Jaipur, Rajasthan 303007, India

Email: dinesh.bhalothia@jaipur.manipal.edu

Prof. Po-Chun Chen

Department of Materials and Mineral Resources Engineering, National Taipei University of Technology

Email: cpc@mail.ntut.edu.tw

Prof. Tsan-Yao Chen

Department of Engineering and System Science,

National Tsing-Hua University, Hsinchu 300, Taiwan

Email: chencaeser@gmail.com;

FAX: +886-3-5720724


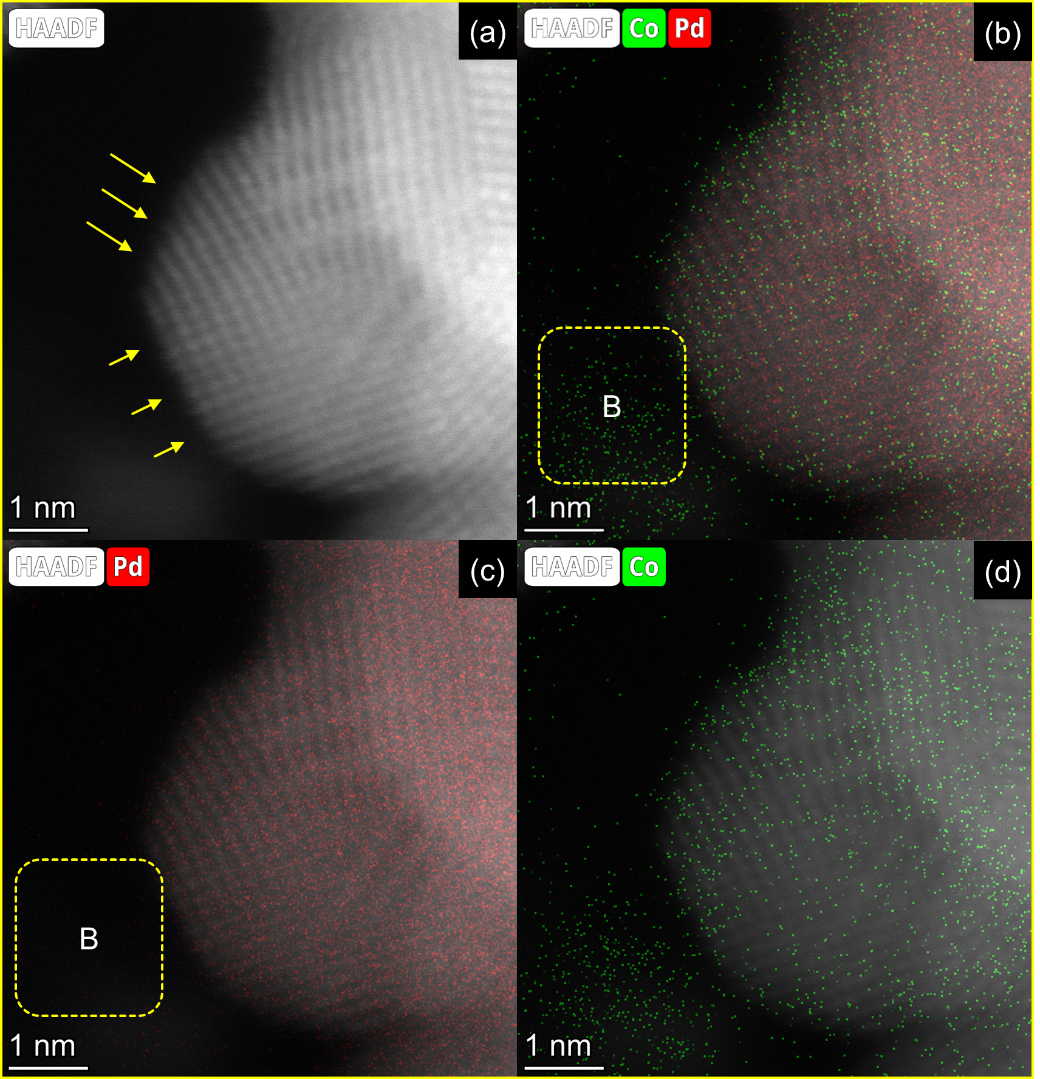


**Figure S1.** The AC-HAADF-STEM image and corresponding EDS elemental maps of Co@Pd catalyst.

**Supplementary note-1:**

For a fair comparison, the HRTEM image of carbon-supported Pd nanoparticles (Pd-BP) has been shown in **Figure S2**, where the Pd-BP exhibits a hazy characteristic on the surface, which can be linked to surface oxidation, resulting in reduced ORR performance. [18] This is further supported by the observed increase in d-spacing to 0.255 nm compared to the 0.225 nm of the Pd (111) plane. **Figure S3** represents the HAADF-STEM image of Co@Pd (i.e. without Ti) with EDS elemental maps of Co and Pd, confirming that Pd nanoparticles are grown on Co-oxide support. Meanwhile, as shown in **Figure S4**, the Co@Pd nanoparticles are grown with severe surface defects/roughness with a typical d-spacing of 0.216 nm. Most importantly, the nearly similar d-spacing of CP@Ti-1 (0.215 nm) as that of Co@Pd along with a relatively smooth surface (**Figure S5**) suggests that the decorated Ti single atoms are accommodated in the surface defects of Pd crystal without disturbing the crystal structure.


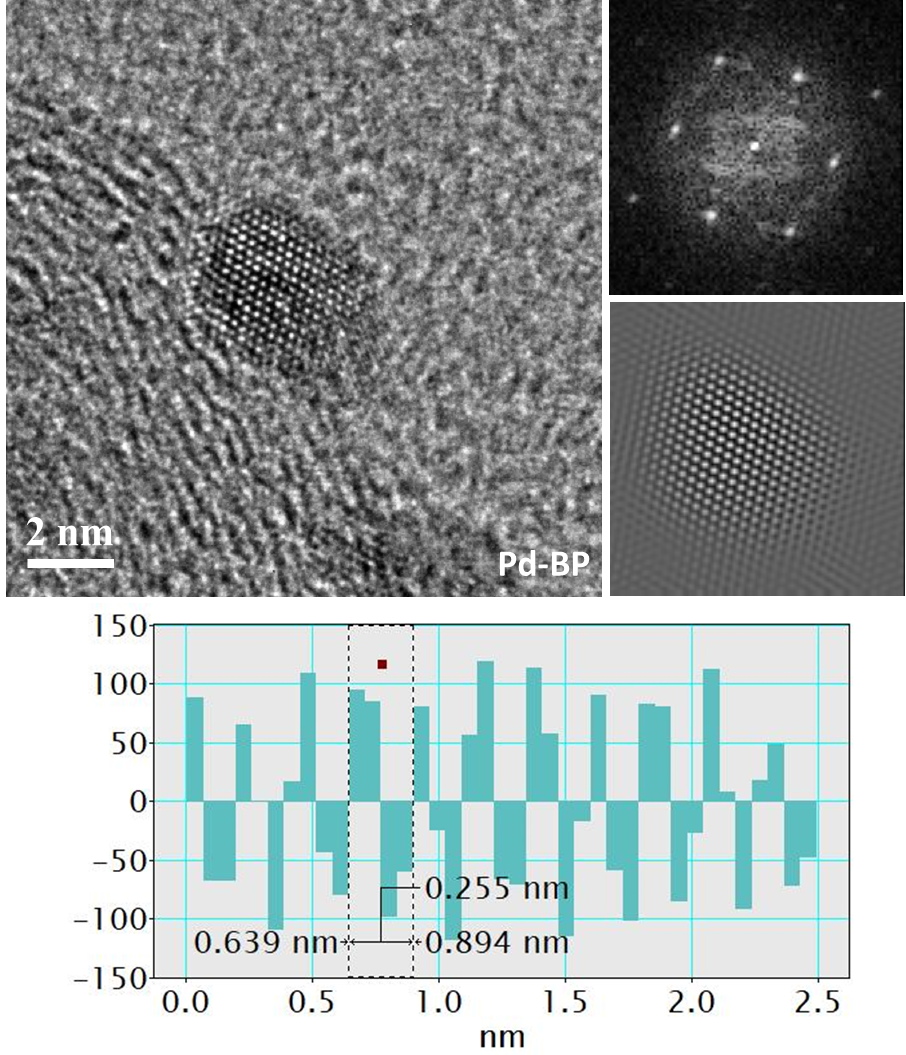


**Figure S2.** The HRTEM image of carbon-supported Pd nanoparticles. The corresponding FFT, IFT patterns and line histogram are shown in the insets.


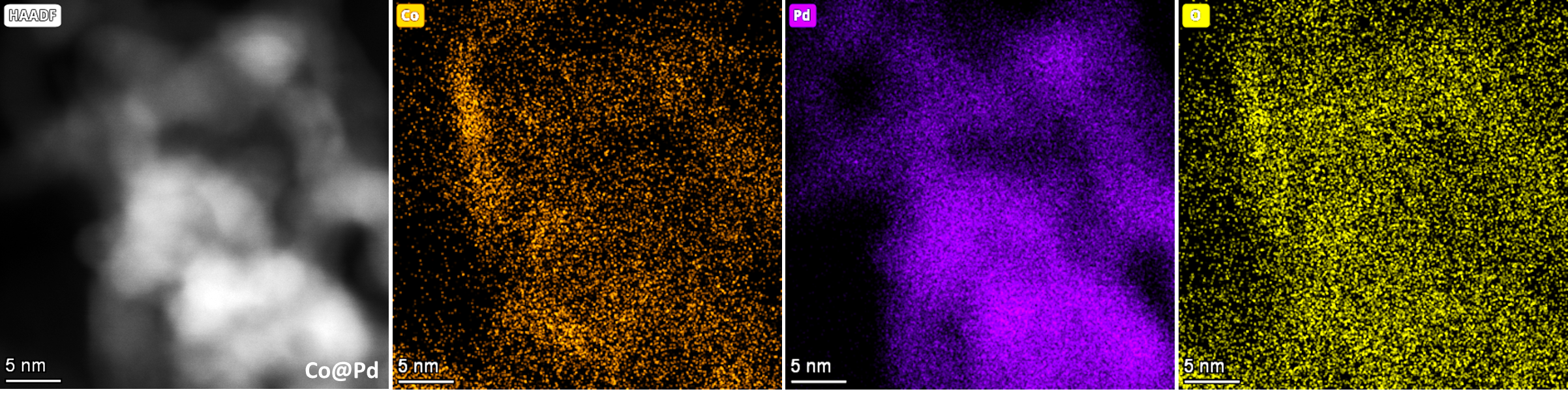


**Figure S3.** (a) The HAADF-STEM image of Co@Pd catalyst. The corresponding EDS elemental maps are shown in the insets.


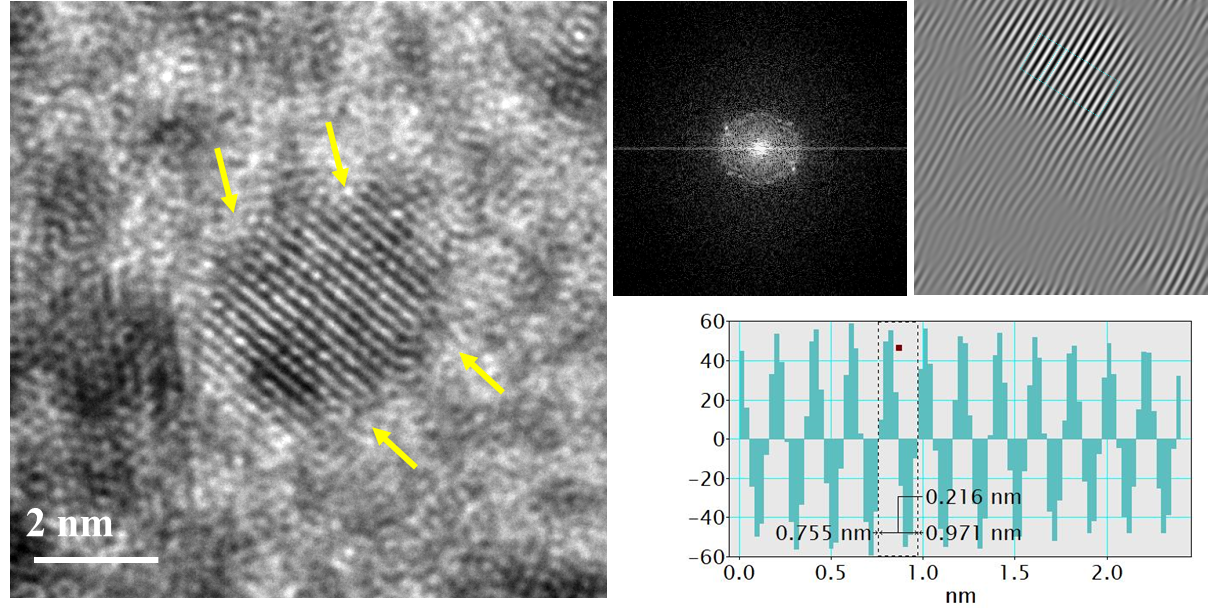


**Figure S4.** The HRTEM image Co@Pd catalyst. The corresponding FFT, IFT patterns and line histogram are shown in the insets.


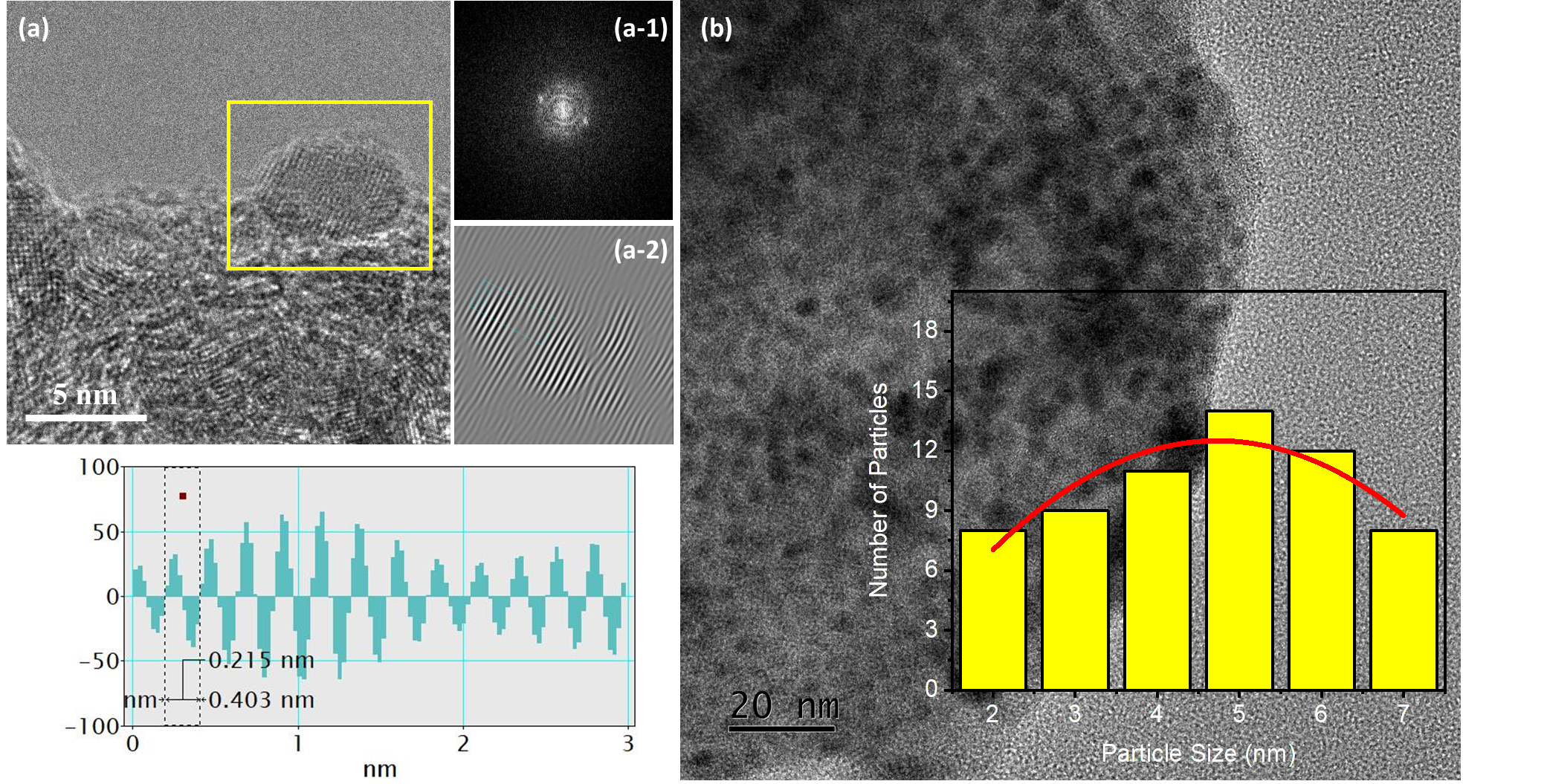


**Figure S5.** (a) The HRTEM image CP@Ti-1 catalyst. The corresponding FFT, IFT patterns and line histogram are shown in the insets. (b) the low resolution TEM image and corresponding particle size histogram.


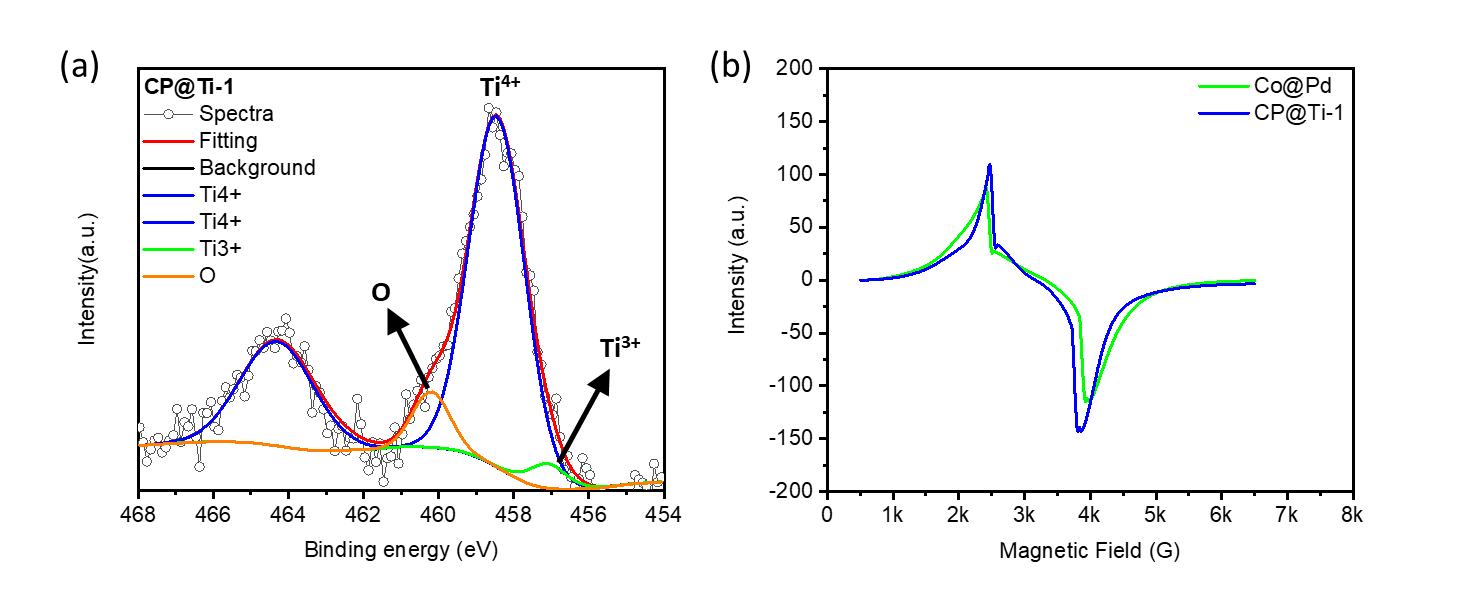


**Figure S6**. (a) The XPS spectra of CP@Ti-1 catalyst at Ti-2p orbital and (b) the EPR spectra of Co@Pd and CP@Ti-1 catalysts.

**Figure S7.** LSV curves of CP@Ti-1 catalyst compared with commercial J.M.-Pt/C catalyst.


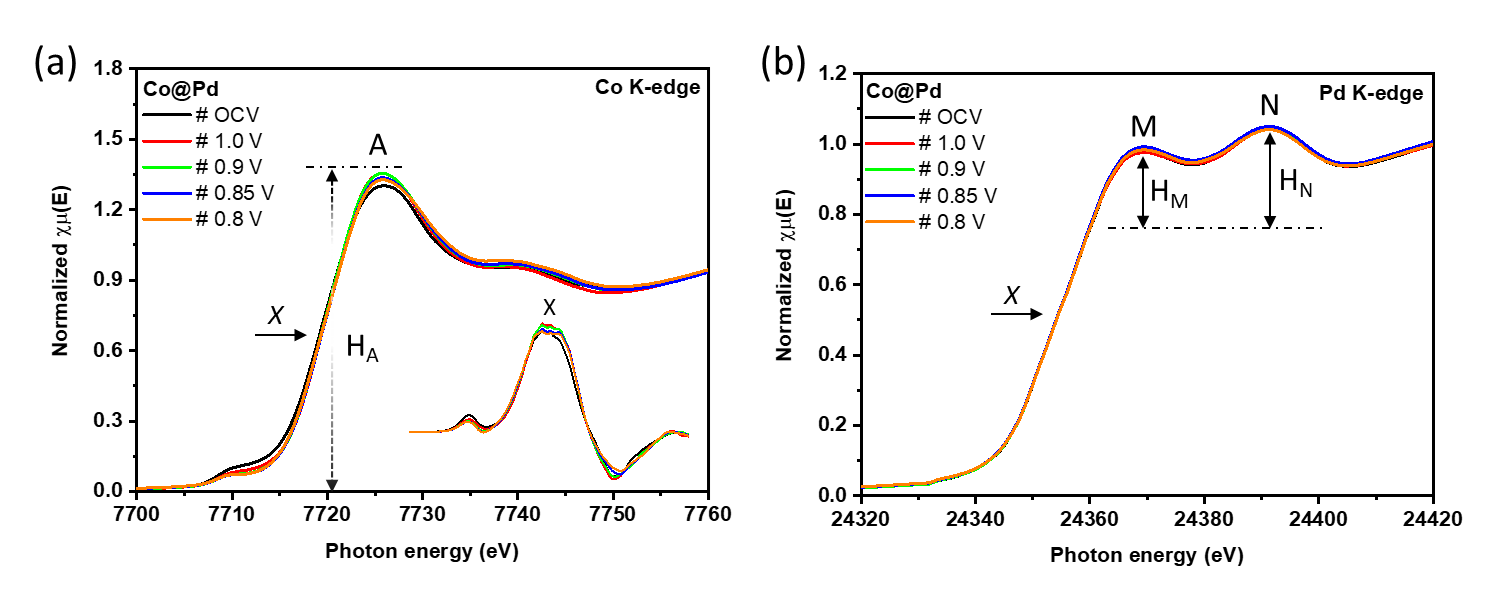


**Figure S8.** (a) In-situ XANES spectra of the Co@Pd (a) Co K-edge and (b) Pd K-edge.

**Supplementary note-2: Calculation procedure for ORR mass activity**

The mass activity (MA) is calculated by following equation

$mass activity (mA mg^{-1})=J_{k}\times\frac{area}{mass of catalyst}$ (S1)

where *J*_k_ is the kinetic current density (mA/cm^2^) and area is the geometric area of working electrode (0.196 cm^2^). The mass activity of the catalyst is estimated via the calculation of *J*_k_ and normalization to the catalyst loading on glassy carbon rotating disk electrode.

It is evident from **Figure 4b** that the carbon-supported Co nanoparticles do not show ORR catalytic activity at 0.85 V vs. RHE, whereas, J_k_ of the Co@Pd is 9.57 mA/cm^2^ (**Figure 4d**). Therefore, with the similar Pd-loading in the CP@Ti-1 catalyst as that of Co@Pd, we deducted the J_k_ of Co@Pd from the original J_k_ of CP@Ti-1 catalyst. In this way, it is reasonable to conclude that the mass activities of the CP@Ti-1 catalyst in **Figure 4e** are dominated by the decorated Ti-single atoms. For easy clarification, the numerical calculation procedures for the MA of the CP@Ti-1 catalyst has been presented below; where the original J_k_ at 0.85 V vs RHE is 25.13 mAcm^-2^ for the CP@Ti-1 catalyst (J_k_(CP@Ti-1)_original_) and 9.57 mAcm^-2^ for Co@Pd. Hence,

J_k_(CP@Ti-1) = J_k_(CP@Ti-1)_Original_ – J_k_(Co@Pd) = 25.13 – 9.57 = 15.56 mAcm^-2^

Now we used 15.56 as J_k_ for the calculation of MA for the CP@Ti-1 catalyst to ensure that MA_Ti_ is completely dominated by the decorated Ti single atoms.

**Table S1. Comparison of ORR performance between NiPP and other Pt-decorated catalysts in 0.1 M KOH electrolyte from literature.**

| **Catalysts** | **E_1/2_ vs RHE** | **V_OC_ vs RHE** | **MA (mAmg_Pt_^-1^)** | | **ADT Cycles** | | **Stability** | | **References** | |
| --- | --- | --- | --- | --- | --- | --- | --- | --- | --- | --- |
|  |  |  | **@ 0.85 V vs RHE** | **@ 0.90 V vs RHE** |  |  |  |  |  |  |
| CP@Ti-1 | 0.884 | 0.934 | 9725 | 1244 | 20 K | | 100% | | This Study | |
| NiPP | 0.883 | 0.94 | 5050.3 | 952.3 | 15 K | | 100% | | 1 | |
| NPP-70 | N/A | | 1009.2 | 484.8 | 10 K | | ∼ 100% | | 2 | |
| Co@Pd/Pt/CNT | 0.88 | 0.944 | 2055.1 | N/A | 310 K | | 100% | | 3 | |
| H-Pt/CaMnO_3_ | 0.81 | 0.95 | 380 |  | 6 K | | 95 % | | 4 | |
| Co@Pt | N/A | | 71.9 |  | 5 K | | 17 mV penalty | | 5 | |
| Pd@PtNi/MWCNT |  |  | 73.3 |  | 5 K | | 100 % | | 6 | |
| Pt-decorated three dimensional N-doped carbon |  |  | N/A | 162.88 | 5 K | | 100 % | | 7 | |
| Pt@Pd nanocubes | 0.88 | 0.97 | 380 | N/A | 1 K | | 80 % | | 8 | |
| Cu@Pd/Pt |  | 0.909 | 414 | N/A | | | | | 9 | |
| PdCu_2_@Pt-H |  | N/A | 485 |  |  |  |  |  | 10 | |
| CPI-SA | 0.883 | 0.94 | 7173 | 770 | | 69 K | | 100% | | 11 |
| Ni@Pd-Ir | 0.861 | 0.91 | 2066 | N/A | | 21 K | | 95% | | 12 |
| ZrN NPs | 0.80 V | N/A | | | | | | | | 13 |
| La0.6Ca0.4CoO3 | ~0.70 V |  |  |  | |  | |  | | 14 |
| Co0.5Mo0.5OyNz/C | 0.76 V |  |  |  | |  | |  | | 15 |
| Cu3P@C | ~ 0.75 V |  |  |  | |  | |  | | 16 |
| TiO2 | < 0.77 V |  |  |  | |  | |  | | 17 |
| NdBa0.7Ca0.25CoxFe2-xO5+δ | < 0.75 V |  |  |  | |  | |  | | 18 |

**Reference**

[1] D. Bhalothia, A. Beniwal, C. Yan, K.-C. Wang, C.-H. Wang, T.-Y. Chen, Potential synergy between Pt2Ni4 Atomic-Clusters, oxygen vacancies and adjacent Pd nanoparticles outperforms commercial Pt nanocatalyst in alkaline fuel cells, Chemical Engineering Journal 483 (2024) 149421. https://doi.org/https://doi.org/10.1016/j.cej.2024.149421.

[2] D. Bhalothia, C. Yan, N. Hiraoka, H. Ishii, Y.-F. Liao, P.-C. Chen, K.-W. Wang, J.-P. Chou, S. Dai, T.-Y. Chen, Pt-Mediated Interface Engineering Boosts the Oxygen Reduction Reaction Performance of Ni Hydroxide-Supported Pd Nanoparticles, ACS Applied Materials & Interfaces 15(12) (2023) 16177-16188. https://doi.org/10.1021/acsami.2c21814.

[3] S. Dai, J.-P. Chou, K.-W. Wang, Y.-Y. Hsu, A. Hu, X. Pan, T.-Y. Chen, Platinum-trimer decorated cobalt-palladium core-shell nanocatalyst with promising performance for oxygen reduction reaction, Nature Communications 10(1) (2019) 440. https://doi.org/10.1038/s41467-019-08323-w.

[4] X. Han, F. Cheng, T. Zhang, J. Yang, Y. Hu, J. Chen, Hydrogenated Uniform Pt Clusters Supported on Porous CaMnO3 as a Bifunctional Electrocatalyst for Enhanced Oxygen Reduction and Evolution, Advanced Materials 26(13) (2014) 2047-2051. https://doi.org/https://doi.org/10.1002/adma.201304867.

[5] L. Wang, Z. Tang, W. Yan, Q. Wang, H. Yang, S. Chen, Co@Pt Core@Shell nanoparticles encapsulated in porous carbon derived from zeolitic imidazolate framework 67 for oxygen electroreduction in alkaline media, Journal of Power Sources 343 (2017) 458-466. https://doi.org/https://doi.org/10.1016/j.jpowsour.2017.01.081.

[6] S. Liu, Y. Wang, L. Liu, M. Li, W. Lv, X. Zhao, Z. Qin, P. Zhu, G. Wang, Z. Long, F. Huang, One-pot synthesis of Pd@PtNi core-shell nanoflowers supported on the multi-walled carbon nanotubes with boosting activity toward oxygen reduction in alkaline electrolyte, Journal of Power Sources 365 (2017) 26-33. https://doi.org/https://doi.org/10.1016/j.jpowsour.2017.08.073.

[7] Y. Cheng, H. Lu, K. Zhang, F. Yang, W. Dai, C. Liu, H. Dong, X. Zhang, Fabricating Pt-decorated three dimensional N-doped carbon porous microspherical cavity catalyst for advanced oxygen reduction reaction, Carbon 128 (2018) 38-45. https://doi.org/https://doi.org/10.1016/j.carbon.2017.10.102.

[8] C.-L. Lee, C.-C. Yang, C.-R. Liu, Z.-T. Liu, J.-S. Ye, Pt-coated Pd nanocubes as catalysts for alkaline oxygen reduction activity, Journal of Power Sources 268 (2014) 712-717. https://doi.org/https://doi.org/10.1016/j.jpowsour.2014.06.112.

[9] H.-Y.T. Chen, J.-P. Chou, C.-Y. Lin, C.-W. Hu, Y.-T. Yang, T.-Y. Chen, Heterogeneous Cu–Pd binary interface boosts stability and mass activity of atomic Pt clusters in the oxygen reduction reaction, Nanoscale 9(21) (2017) 7207-7216. https://doi.org/10.1039/C7NR01224A.

[10] H.-Y. Park, J.H. Park, P. Kim, S.J. Yoo, Hollow PdCu2@Pt core@shell nanoparticles with ordered intermetallic cores as efficient and durable oxygen reduction reaction electrocatalysts, Applied Catalysis B: Environmental 225 (2018) 84-90. <https://doi.org/https://doi.org/10.1016/j.apcatb.2017.11.052>.

[11] D. Bhalothia, C. Yan, N. Hiraoka, H. Ishii, Y. F. Liao, S. Dai, P.-C. Chen, T.-Y. Chen, *Advanced Science* **2024**, *11* (33), 2404076, https://doi.org/https://doi.org/10.1002/advs.202404076.

[12] D. Bhalothia, D.-L. Tsai, S.-P. Wang, C. Yan, T.-S. Chan, K.-W. Wang, T.-Y. Chen, P.-C. Chen, *Journal of Alloys and Compounds* **2020**, *844*, 156160, https://doi.org/https://doi.org/10.1016/j.jallcom.2020.156160.

[13] Y. Yuan, J. Wang, S. Adimi, H. Shen, T. Thomas, R. Ma, J. P. Attfield, M. Yang, *Nature Materials* **2020**, *19* (3), 282, https://doi.org/10.1038/s41563-019-0535-9.

[14] V. Hermann, D. Dutriat, S. Müller, C. Comninellis, *Electrochimica Acta* **2000**, *46* (2), 365, https://doi.org/https://doi.org/10.1016/S0013-4686(00)00593-4.

[15] B. Cao, G. M. Veith, R. E. Diaz, J. Liu, E. A. Stach, R. R. Adzic, P. G. Khalifah, *Angewandte Chemie International Edition* **2013**, *52* (41), 10753, https://doi.org/https://doi.org/10.1002/anie.201303197.

[16] R. Wang, X.-Y. Dong, J. Du, J.-Y. Zhao, S.-Q. Zang, *Advanced Materials* **2018**, *30* (6), 1703711, https://doi.org/https://doi.org/10.1002/adma.201703711.

[17] D.-N. Pei, L. Gong, A.-Y. Zhang, X. Zhang, J.-J. Chen, Y. Mu, H.-Q. Yu, *Nature Communications* **2015**, *6* (1), 8696, https://doi.org/10.1038/ncomms9696.

[18] B. Hua, Y.-Q. Zhang, N. Yan, M. Li, Y.-F. Sun, J. Chen, J. Li, J.-L. Luo, *Advanced Functional Materials* **2016**, *26* (23), 4106, https://doi.org/https://doi.org/10.1002/adfm.201600339.
